# Boosting Zn||I_2_ Battery’s Performance by Coating a Zeolite-Based Cation-Exchange Protecting Layer

**DOI:** 10.1007/s40820-022-00825-5

**Published:** 2022-03-25

**Authors:** Wenshuo Shang, Qiang Li, Fuyi Jiang, Bingkun Huang, Jisheng Song, Shan Yun, Xuan Liu, Hideo Kimura, Jianjun Liu, Litao Kang

**Affiliations:** 1 College of Environment and Materials Engineering, Yantai University, Yantai, 264005 People’s Republic of China; 2 State Key Laboratory of High-Performance Ceramics and Superfine Microstructure, Shanghai Institute of Ceramics, Chinese Academy of Sciences, Shanghai, 200050 People’s Republic of China; 3 Key Laboratory for Palygorskite Science and Applied Technology of Jiangsu Province, Huaiyin Institute of Technology, Huai’an, 223003 People’s Republic of China

**Keywords:** Zeolite, Protecting layer, Zn-I_2_ aqueous battery, Shuttle, Parasitic reactions

## Abstract

**Highlights:**

- High-performance Zn||I_2_ batteries were established by coating zeolite protecting layers.
- The Zn^2+^-conductive layer suppresses I_3_^−^ shuttling, Zn corrosion/dendrite growth.
- The Zeolite-Zn||I_2_ batteries achieve long lifespan (91.92% capacity retention after 5600 cycles), high coulombic efficiencies (99.76% in average) and large capacity (203–196 mAh g^−1^ at 0.2 A g^−1^) simultaneously.

**Abstract:**

The intrinsically safe Zn||I_2_ battery, one of the leading candidates aiming to replace traditional Pb-acid batteries, is still seriously suffering from short shelf and cycling lifespan, due to the uncontrolled I_3_^−^-shuttling and dynamic parasitic reactions on Zn anodes. Considering the fact that almost all these detrimental processes terminate on the surfaces of Zn anodes, modifying Zn anodes’ surface with protecting layers should be one of the most straightforward and thorough approaches to restrain these processes. Herein, a facile zeolite-based cation-exchange protecting layer is designed to comprehensively suppress the unfavored parasitic reactions on the Zn anodes. The negatively-charged cavities in the zeolite lattice provide highly accessible migration channels for Zn^2+^, while blocking anions and electrolyte from passing through. This low-cost cation-exchange protecting layer can simultaneously suppress self-discharge, anode corrosion/passivation, and Zn dendrite growth, awarding the Zn||I_2_ batteries with ultra-long cycle life (91.92% capacity retention after 5600 cycles at 2 A g^−1^), high coulombic efficiencies (99.76% in average) and large capacity (203–196 mAh g^−1^ at 0.2 A g^−1^). This work provides a highly affordable approach for the construction of high-performance Zn-I_2_ aqueous batteries.
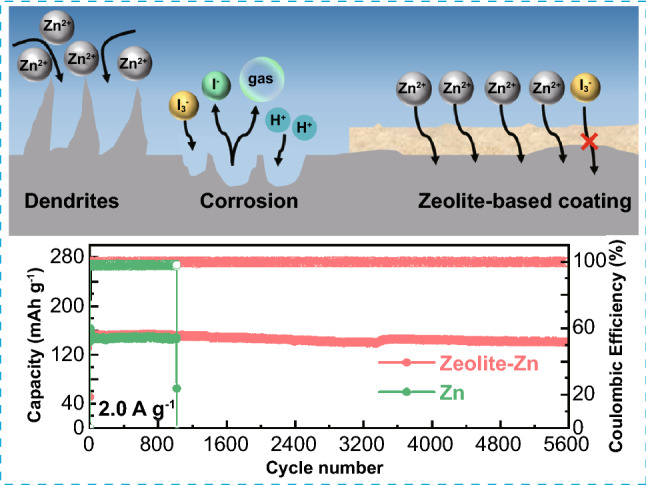

**Supplementary Information:**

The online version contains supplementary material available at 10.1007/s40820-022-00825-5.

## Introduction

While dominating the rechargeable (i.e., secondary) battery market with outstanding energy-/power-density and long lifespan, lithium-ion batteries (LIBs) are still seriously suffering from cost and, especially safety issues [1], due to the use of scarce/high-price elements (e.g., Li, Co) [2] and flammable organic electrolytes [3]. Therefore, the traditional aqueous Pb-acid batteries, despite low energy–density, short-lived and polluting-potential, are still widely adopted in many application scenarios where operational safety and/or cost are the top priorities [4]. With the rapid development of smart grid and large-scale electrochemical energy storage devices, it becomes urgent to develop aqueous batteries that are simultaneously safe, low-cost, green, long-lasting, and high-performance [5].

Significantly, Zn is not only the anode of the historic “voltaic pile,” but also one of the rare metallic anodes successfully commercialized in primary aqueous batteries (e.g., Zn alkaline, Ag-Zn, and Zn-air batteries) [6, 7], thanks to its multifaceted advantages including large theoretical capacity, abundant resource, low-cost, non-toxicity, and high electrical conductivity [8]. Encouraged by the success in primary batteries, numerous attempts have recently been devoted to the development of rechargeable zinc metal batteries (ZMBs) [9, 10]. Nevertheless, converting primary ZMBs into rechargeable is difficult [11], because the repeated Zn striping/plating processes on the anodes dramatically accelerate detrimental parasitic reactions [12], including dendritic Zn deposition [13, 14], surface corrosion/passivation [15], and electrolyte decomposition/consumption [16]. Furthermore, many intercalation-type ZMBs’ cathodes are unstable in the aqueous electrolytes [17], due to byproduct-derived surface passivation [18] and/or electrolyte etching [19–21].

Remarkably, iodine (I_2_) cathode stores electrons through the direct conversion reaction between solid I_2_ and soluble I^−^ anions, providing a significant theoretical capacity of 211 mAh g^−1^ [22, 23]. This reaction does not generate irreversible byproduct, thus is highly reversible and virtually passivation-free [24]. Even when the I_2_ is etched or reduced by specific component in the electrolytes, the resulting I^−^ species can still contribute capacity by oxidizing back to I_2_ in following charge process, thanks to its high solubility and proper redox potential [25, 26]. The major problems of this affordable cathode lie on the low electrical conductivity of I_2_, as well as the formation of soluble triiodide (i.e., I_3_^−^) intermediate species via I_2_/I^−^ complexing (I_2_ + I^−^ → I_3_^−^) [27, 28]. The I_3_^−^ dissolving in electrolyte can easily penetrate through routine glass fibers (GFs) [29] or polypropylene [30] separators, and quickly react with the metallic Zn anode (by I_3_^−^ + Zn → Zn^2+^  + I^−^), leading to fast I_2_ loss and self-discharge [23, 31].

To restrain the free migration of I_3_^−^, the I_2_ active materials are usually confined into porous matrix with high adsorption capability (e.g., active carbon [28–30, 32] and MXene [33]) and even electrocatalytic ability (Co/Fe-hexacyanoferrate [27]). The nano-pore confining design restrains both I_3_^−^ generation and migration, leading to much-improved cycle and shelf life [27, 29]. At the same time, the AC (active carbon) and MXene matrix contribute not only additional capacity, but also prominent electrical conductivity, ensuring high I_2_-utilizing efficiencies even at high-rate charge/discharge [28]. Moreover, manipulating the electrolytes with novel zinc salts [29] or immobile anionic gelatinizing skeletons [34] prove also effective to suppress the I_3_^−^-shuttling from cathode to anode, by means of modulating either coordination [29] or electrostatic repulsion between I_3_^−^ and the electrolyte [34], respectively. In the well-established Zn||I_2_ flow batteries, the famous nafion cation-exchange membranes, as benchmark commercial separators, are usually employed to suppress the crossover migration of I_3_^−^ anions [35]. Unfortunately, nafion membranes are, currently, too expensive to maintain the cost competitiveness of the Zn||I_2_ batteries, even being robust and durable. To circumvent this dilemma, Zhou’s group invented an artful ionic-sieve membrane separator based on Zn-BTC metal organic framework (MOF) [23]. This MOF separator can block not only I_3_^−^ shuttling, but also parasitic reactions by regulating the electrolyte solvation structure.

In Zn||I_2_ batteries, almost all the parasitic reactions terminate on the surfaces of Zn anodes [16, 17]. Therefore, modifying Zn anode’s surface with protecting coatings should be one of the most straightforward and thorough approaches to synchronously restrain these detrimental processes [13, 36, 37]. An adequate protecting coating can virtually isolate Zn anodes from the aqueous electrolytes [38], effectively suppressing Zn corrosion/passivation, H_2_ evolution and electrolyte consumption that associate with the reaction between Zn and electrolyte [16, 39], as well as the quick self-discharge caused by reactions between I_3_^−^ and Zn [40]. It means that the coating should be able to selectively block I_3_^−^ while smoothly conducting Zn^2+^ [23]. In other words, the coating should have a strong cation-exchange ability, in order to simultaneously achieve excellent charge/discharge performance and long shelf-life (i.e., low self-discharge rate) [22]. This is also the exact reason why nafion and MOF membrane separators have been used in Zn||I_2_ batteries [23, 35].

In this contribution, we propose a high performance and low-cost rechargeable Zn||I_2_ secondary batteries with cheap aqueous ZnSO_4_ electrolytes, by protecting the Zn anode with a zeolite-based cation-exchange coating. Zeolite, famous as the oldest molecular sieve, is a series of important inorganic microporous minerals featured with high cationic conductivity, low electronic conductivity and excellent stability, thanks to its unique aluminosilicate open framework [41]. In these materials, the replacement of some [SiO_4_] tetrahedra by [AlO_4_] imposes cavities and negative charges to the lattice framework, allowing the accommodation of mobile cations (such as zinc, lead, and cadmium) in the cavities. At the same time, the negatively-charged cavities can electrostatically forbid anions to pass through, achieving a precious cation-exchange ability at very low cost [42]. By simply coating a zeolite-based layer on Zn anode, the obtained Zeolite-Zn||I_2_ batteries simultaneously achieved large capacity (196 mAh g^−1^ at 0.2 A g^−1^), high coulombic efficiencies (99.76 and 98.53% in average at 2 and 0.2 A g^−1^, respectively), excellent cycling durability (91.92% capacity retention after 5600 cycles at 2 A g^−1^, capacity decay rate: 0.0016% per cycle), and long shelf life (83% capacity retention after 50 h static resting). Compared with currently available strategies, this approach shows outstanding advantages in cost and environmental benignity, while delivering comparable performance. Moreover, density functional theory calculation suggests that microstructural optimization may be able to further improve the effectiveness of this strategy.

## Experimental and Caculation

### Materials Preparation and Device Assembly

#### Preparation of Zn-Based Zeolite and Zn-Based Zeolite Coated Zn Foil (Zeolite-Zn)

The commercial artificial zeolite (average size: ~ 10 μm) was provided by Shanghai Aladdin Bio-Chem Co., Ltd. XRD assessment indicates the powder is a mixture of the FAU framework (JCPDS No. 38–0241) and ETR framework type zeolite (JCPDS No. 71–1557, Fig. S1). To prepare the Zn^2+^-exchanged zeolite, 1 g pristine zeolite was added into 80 mL deionized (DI) water containing 0.5 g ZnSO_4_ (corresponding to a concentration of 1 M, AR grade, Aladdin Bio-Chem Co., Ltd), and mechanically stirred for 6 h. After thoroughly washing and drying, the Zn^2+^-exchanged zeolite powder was mixed with polyvinylidene difluoride (PVDF, weight ratio: 8:2, as binder) in proper amount of N-methyl pyrrolidone (NMP) solvent by grinding. The resulting slurry was uniformly coated on bare Zn foils and dried at 60 ℃ overnight. The zeolite/PVDF coated Zn samples were named as Zeolite-Zn below.

#### Preparation of the I2@AC Composite

The I_2_@AC composite cathode was prepared via an I_2_ sublimation method [28, 29]. Briefly, 0.5 g I_2_ and 0.5 g activated carbon (AC) were thoroughly mixed by grinding. Afterward, the mixed powder was sealed in a hydrothermal reactor and heated at 90 ℃ for 4 h. During heating, the I_2_ was thermally sublimated and infused into the pores of the activated carbon. After natural cooling, the porous carbon enveloped I_2_ (I_2_@AC) composite was obtained (Fig. S2).

#### Preparation of I_2_@AC Cathode Electrode

The cathode coating slurry was fabricated by mixing the I_2_@AC powders, acetylene black (conductive agent) and polyvinylidene difluoride (PVDF, binder) at a weight ratio of 7:2:1 with proper amount of N-methyl pyrrolidone (NMP) as solvent. The resulting slurry was uniformly coated on graphite paper (GP, current collector) and dried at 40 ℃ for 12 h. The I_2_@AC-coated GP was cut into Φ16 mm discs and used as cathodes for further Zn||I_2_ battery assembly. To clarify whether or not the I_2_ in the I_2_@AC can be dissolved by the NMP solvent, we collected the TG curves of the dried slurry in a dynamic nitrogen atmosphere within 30–800 °C (Fig. S3). Up to 300 °C, the dried slurry demonstrates a I_2_ sublimation weight loss of 33.62%, in good line with the theoretical value of 35.00% (I_2_@AC: acetylene black: PVDF weight ratio of 7:2:1, 50% I_2_ in the I_2_@AC powder). This TG curve evidently confirms the survival of the I_2_ from dissolution during electrode preparing process, thanks to the strong interactions between the AC matrix and the infused I_2_ (see Fig. S2e, pay attention that the I_2_ transformed from crystalline into amorphous after loading into the AC matrix). Above 300 °C, the 8.94% weight loss between 310 and 500 ℃ can be attributed to the decomposition of PVDF [43]. We also prepared pure AC cathodes following the same preparation process, in order to determine the capacity contribution from the AC component.

#### Zn||Zn Symmetrical Cell Assembly

Bare- and Zeolite-Zn foils (50 μm in thickness) were firstly cut into Φ16 mm discs. Then, two Zn discs (bare- or Zeolite-Zn) were separated by a glass fiber paper (Φ = 19 mm) thoroughly wetted by 1 M ZnSO_4_ electrolyte, and then packed into a 2032-type button cell. To standardize the measurement, a fixed amount (120 μL) of electrolyte was used in each coin cell. All batteries were assembled in ambient air atmosphere.

#### Zn||I_2_ Battery Assembly

The Zn||I_2_ batteries were assembled with the GP-supporting I_2_@AC discs as cathodes, glass fiber papers as separators, and Zeolite-Zn (or bare-Zn) discs as anodes, in a form of CR2032 coin cell. The assembling processes were completed in ambient air atmosphere, with a 1 M ZnSO_4_ aqueous electrolyte.

### Material Characterization

X-ray powder diffraction (XRD) was carried out on a D/max-2500/PC X-ray diffractometer with Cu Kα radiation (*λ* = 0.15418 nm). The surface morphology and element mapping of the samples were characterized by a JEOL JSM-7610F field emission scanning electron microscope (SEM) equipped with an energy-dispersive spectroscope (EDS). The electrolyte/anode contact angles were measured by an optical contact angle and interface tension meter (CA, CA100C, Innuo, China) at room temperature in air, and a 10 μL droplet of 1 M ZnSO_4_ electrolyte was used in the experiment. Thermogravimetry analysis (TGA) was carried out on a Shimadzu TGA-60 analyzer, within 30–700 °C in a dynamic nitrogen atmosphere (100 mL min^−1^). In this test, the heating rate is fixed to 10 °C min^−1^. The UV absorption spectra of different electrolytes were measured on a Persee TU-1810 UV–Vis spectrophotometer. The Brunauer–Emmett–Teller (BET) specific surface areas of the AC and I_2_@AC powders were determined by N_2_ sorption method on an ASAP 2460 physical adsorption analyzer (Micromeritics, America) at 77.3 K. Before BET testing, the samples were outgassed in a vacuum at 120 °C for 2 h. The pore size distributions were derived from the adsorption branch using the Barrett-Joyner-Halenda (BJH) model.

### Electrochemical Measurements

Galvanostatic charge–discharge (GCD), Coulombic efficiency (CE) and rate capability tests of all cells were performed on a LAND-CT2001A battery-testing instrument. The Zn||Zn symmetrical cells were GCD cycled at a current density of 2.5 mA cm^−2^ with an areal capacity of 2.5 and 10 mAh cm^−2^ (corresponding to 10 and 40% depth of discharge, DOD, respectively). Bare-Zn||bare-Cu and Zeolite-Zn||Zeolite-Cu cells were also assembled to explore the influence of the zeolite-based coating on Coulombic efficiency (CE, the ratio of Zn stripping capacity to plating capacity). The Zn||I_2_ battery was cycled in a GCD manner between 0.5 and 1.6 V at either 0.2 or 2 A g^−1^ (1C = 211 mAh g^−1^). Cyclic voltammetry (CV), electrochemical impedance spectroscopy (EIS), potentiostatic polarization and Tafel curves were collected on a CHI-660E electrochemical workstation in a two-electrode configuration. The EIS spectra were collected within a frequency range of 10^–2^–10^6^ Hz under a bias of 10 mV (vs. Zn/Zn^2+^). To determine the specific ionic conductivity contributed by Zn^2+^ ion, a potentiostatic polarization method was utilized with a 10 mV bias applied to Zeolite-Zn/Zeolite-Zn and Zn/Zn symmetric cells. In the Tafel test, a Zn foil was used as the working electrode, while another Zn foil as the counter/reference electrode.

### Computational Methods

The positions occupied by atoms in the crystal structure of the substances in this study were generated by the Supercell Program [44]. For fractional occupation as well as fractional occupation structures, the ten structures with the lowest Coulomb energy were obtained by random sampling and Coulomb energy calculation. And the substances were screened using the original cell structure; the experimentally obtained lattice parameters were used to construct the solid-state electrolyte structures. All first-principles calculations are performed with the projector augmented wave (PAW) potential [45, 46] and the Vienna Ab initio Simulation Package (VASP) [47]. The structures screened by Supercell Program were subjected to structural relaxation using VASP software to take the lowest energy structures. Structural relaxation was achieved with a total energy of 10^–5^ eV and a force of 0.01 eV Å^−1^ as convergence criteria. The truncation energy is uniformly set to 520 eV during the calculation.

## Results and Discussion

### Characterization of the Zeolite-Based Protecting Layers

**Fig. 1:**
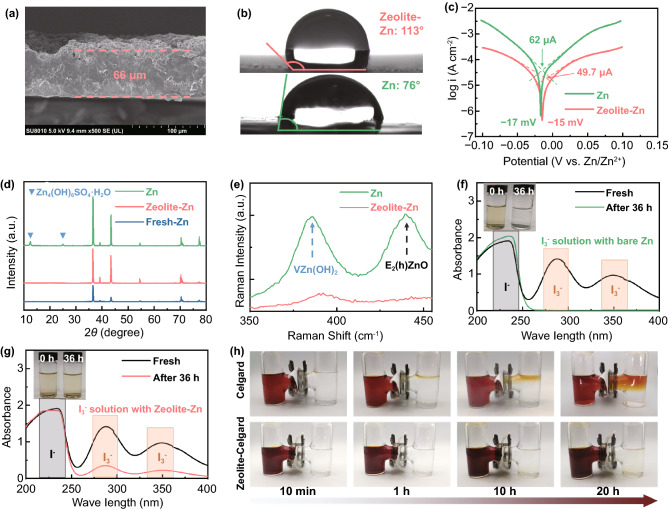
**a** A typical cross-sectional SEM image of the zeolite protecting layer. **b** Contact angles of 1 M ZnSO_4_ electrolyte on either a bare- or Zeolite-Zn foil. **c** Tafel curves of a bare- and Zeolite-Zn foil in the electrolyte. **d** XRD patterns and **e** Raman spectra of the bare- and Zeolite-Zn foil after 15 days static corrosion in 1 M ZnSO_4_ electrolyte. **f-g** UV–vis absorption spectra of the I_3_^−^ electrolytes before and after soaking a bare- or Zeolite-Zn disc for 36 h. **h** Photographs showing the I_3_^−^-shuttling rate across a bare and zeolite-coated commercial Celgard separator. The right and left tanks of the H-shape container were filled with deep brown triiodide solution (i.e., 0.1 M KI + 0.1 M I_2_, left tank) and colorless 0.1 M KI solution (right tank), respectively

Before coating, the pristine zeolite powder underwent a Zn^2+^-exchange treatment in 1 M ZnSO_4_ for 6 h, in order to provide immediate Zn^2+^ supply for smooth Zn stripping/plating (Fig. S1a-b) [35, 48]. Afterward, the zeolite-based protecting layers were deposited on Zn foils via a simple knife coating method, with a blend slurry of the Zn^2+^-exchanged zeolite and PVDF binder (weight ratio: 8:2) in NMP solvent. As revealed by SEM observation, the layers are very dense in the vertical direction, without any detectable penetrating holes/cracks (Fig. 1a and S4a-b), even exhibiting a rough top surface (Fig. S4c-d). The large surface roughness [49, 50] and the hydrophobic nature of the PVDF binder [51] endow the zeolite-Zn a much larger contact angle (CA) than the bare Zn foil (113° vs. 76°, Fig. 1b), very favorable to isolate the underneath Zn foil from the corrosive aqueous electrolyte. As a result, the corrosion potential of the Zn foils shifts from − 17 to − 15 mV after zeolite-layer coating, along with a lower corrosion current (from 62 to 49.7 μA, Fig. 1c), indicating a suppressed corrosion rate on the Zeolite-Zn foils [13, 52].

To further confirm the anticorrosive ability of the protecting layer, a static self-corrosion experiment was performed by soaking a bare- and Zeolite-Zn foil in 1 M ZnSO_4_ electrolytes for 15 days. Postmortem XRD, Raman and SEM analyses (Figs. 1d–e and S5a-c) clearly show the formation of Zn_4_(OH)_6_SO_4_·5H_2_O and even ZnO on the surface of the soaked bare-Zn [15, 16, 23]. On the other hand, the generation of corrosion products and the surficial morphology evolution of the underneath Zn are remarkably suppressed by the zeolite-based protecting layer (Fig. S5d-f), suggesting a slow corrosion rate. Probably, the anticorrosive ability of the layer stems from not only the physical isolation of electrolyte from Zn anodes, but also the desolvation of hydrate Zn^2+^ by the zeolite cavities. As revealed by first principles calculation (Fig. S6), the lowest migration barriers of bare and hydrate Zn^2+^ in the FAU-/ETR-type zeolites are determined to be 0.0086/0.3821 and 0.5002/1.4648 eV, respectively, indicating the much higher migration difficulty of hydrate Zn^2+^ than its bare counterparts. Therefore, the pores of FAU and ETR zeolite are helpful to remove the solvation sheath of hydrate Zn^2+^, due to the very different migration barriers between the bare and hydrate Zn^2+^.

Based on theoretical calculation, we also investigate the transport behaviors of I_3_^−^ ions within the zeolite lattice. The migration barriers of I_3_^−^ are 0.59/1.60 eV in FAU-/ETR-type zeolite, respectively (Fig. S7), much higher than those of the bare and hydrate Zn^2+^. The even harder migration of I_3_^−^ indicates that the zeolite layer can not only protect Zn from water-induced corrosion, but also suppress the quick self-discharge caused by I_3_^−^ shuttling. The quite different migration barriers in different zeolites further highlight the significant influence of zeolites’ structure on cation sieving and Zn^2+^ desolvation. This topic is worthy for additional in-depth study.

To experimentally examine the I_3_^−^-blocking ability of the zeolite-based coating, we specifically designed a spectrophotometry test by making good use of the characteristic optical absorption (centered at 288 and 354 nm) and intense brown color of this anion [25]. Firstly, I_3_^−^-pregnant ZnSO_4_ electrolyte (with 0.5 mM KI + 0.5 mM I_2_) was employed to simulate the electrolyte in real Zn||I_2_ batteries [23]. Then, identically-sized Zeolite-Zn and bare-Zn foils were separately immersed into the I_3_^−^-pregnant solution. After 36 h, the brown color of the bare-Zn soaking solution, along with the absorption bands of I_3_^−^, had thoroughly faded, suggesting the completely reduction of I_3_^−^ by metallic Zn (Fig. 1f). In contrast, considerable amount of I_3_^−^ survived in the Zeolite-Zn soaking solution, as revealed by the brown color and the relatively strong residual absorption bands of I_3_^−^ (Fig. 1g), indicating the remarkable I_3_^−^-blocking ability of the zeolite protecting layer. Moreover, the I_3_^−^-blocking ability of the zeolite layer can also be confirmed by another visually-monitored shuttling experiment, as shown in Fig. 1h [53]. In the H-shape quartz container, the left and right chambers are filled with brown I_3_^−^ (0.1 M KI and 0.1 M I_2_) and colorless ZnSO_4_ solutions, respectively. These chambers are severed by either a bare or a zeolite-coated commercial Celgard separator. With a bare Celgard separator, the colorless ZnSO_4_ solution in the joint-neck position of the left chambers turns to light yellow after only 10 min. Afterward, the color gradually deepens into orange-brown and occupies the whole upper layer of the solution, due to the fast penetration of I_3_^−^ through the porous separator [23]. In striking contrast, the chamber isolated by the zeolite-coated Celgard separator keeps nearly colorless after even 20 h, thanks to the suppression of I_3_^−^-shuttling by the protecting layer.

**Fig. 2:**
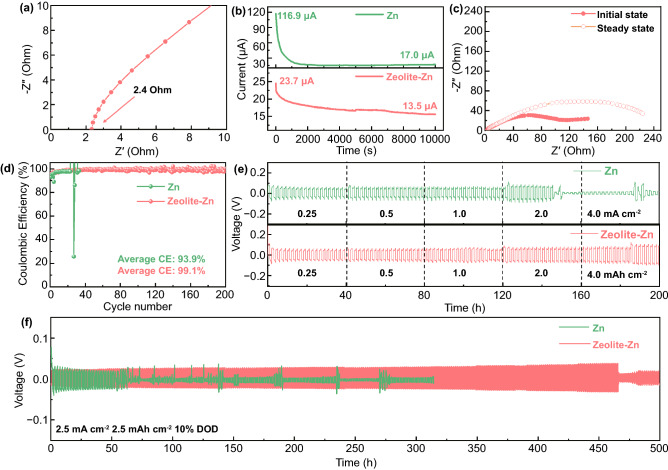
**a** EIS plot of the zeolite layer. **b** Current–time (I-t) curves of a Zn||Zn and Zeolite-Zn||Zeolite-Zn symmetric cells stimulated by a constant polarization voltage of 10 mV. **c** EIS plot of the symmetric cells before (initial state) and after applying voltage polarization for 10,000 s (steady state). **d** Coulombic efficiencies (CEs) of a bare Zn||Cu and a Zeolite-Zn||Zeolite-Cu asymmetric cells in 1 M ZnSO_4_ electrolyte; the employed current density is 0.5 mA cm^−2^ with a striping upper-limit voltage of 0.5 V. **e–f** Voltage profiles of Zn||Zn and Zeolite-Zn||Zeolite-Zn symmetric cells during galvanostatic cycling test in 1 M ZnSO_4_ electrolyte

Besides high anticorrosive and I_3_^−^-blocking performance, an adequate Zn-anode protecting layer needs also a high Zn^2+^ ionic conductivity to ensure smooth Zn striping/plating on the anodes. To determine this property, a free-standing zeolite layer is sandwiched between two stainless steel current collectors after electrolyte wetting. With the thickness and EIS data (electrochemical impedance spectra, Fig. 2a), the ionic conductivity (σ) of the wetted zeolite layer was calculated to be 1.4 mS cm^−1^, according to Eq. (1) [54]:

1 $$\sigma = \frac{l}{{{\text{RS}}}}$$

where *R* represents the equivalent series resistance determined by EIS measurement (2.4 Ω in this case), and *l* and *S* represents the layer thickness (66 μm) and area (2 cm^2^), respectively.

It is worth noting that, the EIS-deduced ionic conductivity is a total value contributed by all the charge carriers, including both cations and anions [54]. To determine the specific conductivity contribution by Zn^2+^, the transference numbers of Zn^2+^ (TZn^2+^) were tested by combining potentiostatic polarization and EIS measurements (Fig. 2b–c) [48, 55]. In the potentiostatic polarization test, the bias voltage (Δ*V* = 10 mV) stimulates a large initial current (I_0_) at the beginning, which gradually decreases to a smaller steady-state current (I_ss_) due to the establishment of stable concentration polarization in the vicinity of the electrode. With the assistance of EIS-determined charge transfer impedances (R_0_, R_ss_, Fig. S8), the Zn^2+^ transference number (TZn^2+^) of the protecting layer can be calculated following Eq. (2) [56]:

2 $$T_{{Zn^{2 + } }} = \frac{{\frac{\Delta V}{{I_{0} }} - R_{0} }}{{\frac{\Delta V}{{I_{ss} }} - R_{ss} }}$$

Compared to the Zn||Zn symmetric cell, the Zeolite-Zn||Zeolite-Zn cell is small in both I_0_ (23.7 vs. 116.9 μA) and I_ss_ (13.5 vs. 17.0 μA, Fig. 2b), because of the suppression of anion-contributed ionic conductivity [54]. As a result, the T_Zn_^2+^ of the symmetric cells is significantly improved from 0.15 to 0.53 by the zeolite layer. The simultaneous achievement of high ionic conductivity and large Zn^2+^ transference number indicate that the zeolite layer is well-performed in both Zn^2+^-conducting and I_3_^−^-blocking [48]. In addition, the layer’s excellent anti-corrosive ability awards the Zeolite-Zn electrodes high striping/plating reversibility, as shown by their higher Columbic efficiency (99.1% vs. 93.9% in average) and much longer cycling lifetime (200 vs. 29 cycles) than the Zn electrodes (Figs. 2d and S9).

The dramatically improved striping/plating stability of the Zeolite-Zn electrodes can also be confirmed by the galvanostatic charge/discharge (GCD) cycling test. As shown in Fig. 2e, the Zn||Zn symmetric cell failed after ~ 140 h during test with stepwise increasing current densities. While the Zeolite-Zn cell easily passed through the 200 h test, with only a minor increase in overpotential from 67.0 to 94.8 mV (Fig. S10), thanks to the high ionic conductivity of the protecting layer. At a relatively large DOD (depth of discharge) of 10% (Eq. S1), the zeolite-based protecting layer dramatically prolongs the lifetime of the symmetric cells by more than 7 times (from 64 to 460 h), while remaining the overpotentials comparable (Figs. 2f and S11). Fitting results of bare- and Zeolite-Zn electrodes’ EIS plots indicate that the Zeolite-based coating slightly elevates the electrode’s equivalent resistance (*R*_s_) from 1.41 to 2.32 Ω cm^−2^, due to electric insulation of the layer (Fig. S12, Table S1). At the same time, the coating also reduced the charge-transfer resistance (*R*_ct_) from 157.26 to 140.50 Ω cm^−2^, probably because of the down-sized Zn nuclei and the modified Zn striping/plating kinetics [14, 52]. The competition between enlarged *R*_s_ and reduced *R*_ct_ results in the comparable overpotentials. At even an ultrahigh DOD of 40%, the lifetime of the Zeolite-Zn cell is still overwhelmingly longer than its bare-Zn counterpart (280 vs. 48 h, Fig. S13). The capability of cycling at high current density and large DOD is crucial for the achievement of high power and energy outputs.

**Fig. 3:**
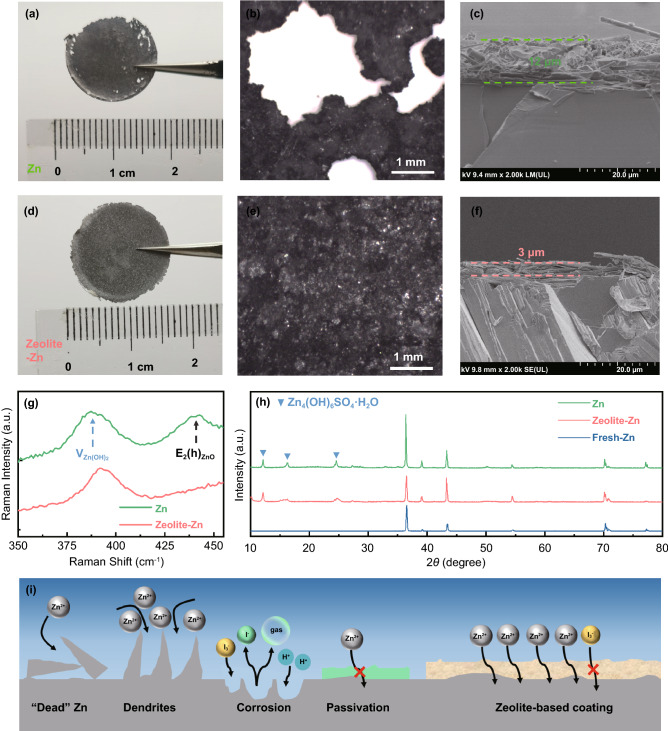
**a, d** Photographs, **b, e** optical micrographs, **c, f** SEM images, **g** Raman spectra and **h** XRD patterns of the bare-Zn and Zeolite-Zn electrodes after working for 313 h and 500 h at 2.5 mA cm^−2^ and 2.5 mAh cm^−2^ in the symmetric cells, respectively. **i** Schematical illustration showing the protecting effects of the zeolite-based layers

To explore the reason why the Zeolite-Zn electrodes are more long-lasting, the cycled bare- and Zeolite-Zn electrodes were disassembled from the asymmetric cells, and thoroughly rinsed to remove the attached electrolyte salt and GF separator. The fragmentary and holey edge of the cycled bare-Zn electrode, along with its rough concertina-like surficial morphology, evidently reveal the dynamic redistribution of Zn caused by the uneven dendritic deposition (Fig. 3a–c and S14a-b) [14]. In addition, the repeated striping processes may break some Zn dendrites at their roots, resulting in many isolated “dead” Zn debris losing capacity contribution (Fig. S15) [57, 58]. During cyclic test, the accumulation of both Zn dendrites and debris on the bare Zn electrode pierce though batteries’ separators and provoke short-circuit failure [57, 59]. In contrast, the cycled Zeolite-Zn electrode remains a decent shape integrity, as well as a compact surficial morphology without any detectable holes or isolated fragments (Fig. 3d–f and S15c-d). In fact, the zeolite/PVDF composite protecting layers should be able to profoundly modify Zn striping/plating behaviors through multiple mechanisms [14], including uniformizing Zn^2+^ flux, increasing deposition nuclei density, downsizing deposits’ dimension, confining deposition position and even dielectric effect [60, 61]. Furthermore, the zeolite-based coating can also suppress the electrolyte-induced corrosion and surface passivation [16, 17], as evidenced by the weak XRD and Raman signals of the corrosion products (e.g., ZnO, Zn(OH)_2_, and Zn_4_(OH)_6_SO_4_·H_2_O, Fig. 3g–h). In short, the multifunctional Zeolite-Zn electrodes perform simultaneously well in anti-corrosion, I_3_^−^-blocking, Zn^2+^-conducting, and dendrite suppression (Fig. 3i).

### Electrochemical Performance of Zn||I_2_ Batteries

**Fig. 4:**
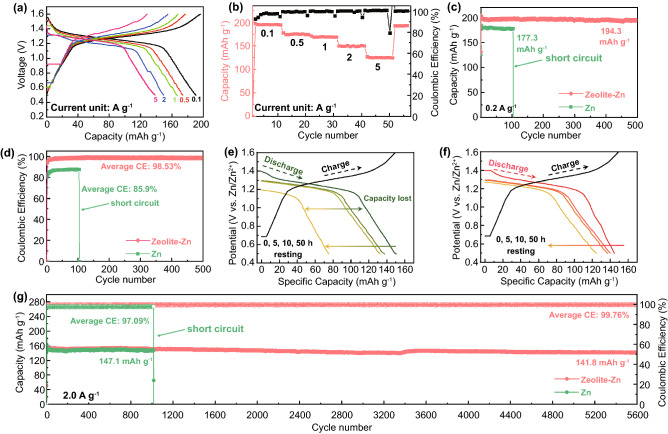
**a** GCD curves and **b** rate performance of the Zeolite-Zn||I_2_ battery. **c** Cycling performance and **d** Coulombic efficiencies (CEs) of the Zn||I_2_ batteries with either bare- or Zeolite-Zn at 0.2 A g^−1^. **e**–**f** Electrochemical aging test (static resting after fully charge state) of Zn||I_2_ batteries with either bare-Zn or Zeolite-Zn anode. **g** Capacity and CE evolution of Zn||I_2_ batteries at current density of 2 A g^−1^ with either bare-Zn or Zeolite-Zn anode

The outstanding performance of the zeolite-based protecting layers encourages us to further explore their influence on Zn||I_2_ full batteries. As shown in Fig. 4a–b, the battery with a Zeolite-Zn anode delivers a high capacity of 195 mAh g^−1^ at 0.1 A g^−1^, along with a very small polarization voltage of 54 mV (Fig. S16) (i.e., voltage difference between charge and discharge plateaus), thanks to the high ionic conductivity of both the protecting layer and aqueous electrolyte. At an ultrahigh current density of 5 A g^−1^, the capacity still reaches up to 125.4 mAh g^−1^, indicating an excellent rate capability (Fig. 4a–b). Pay attention that the capacities of the I_2_@AC cathodes are based on the weight of I_2_, considering the low-capacity attribution from the AC component (~ 8% at 0.2 A g^−1^, Figs. S17 and 4c). This capacity is convenient to reflect the utilizing efficiency of the I_2_ redox reactions, and therefore is widely adopted by different researchers [28, 29, 62]. In fact, the capacity contribution from AC component should be even lower than the tested value, due to I_2_ loading and coverage.

Figure 4c–d further depicts influence of the zeolite protecting layer on the capacities and CEs of Zn||I_2_ full battery. At 0.2 A g^−1^, the bare-Zn anode battery delivers a decent and stable capacity of ~ 178.1 mAh g^−1^, thanks to the excess-mass design of the Zn anode [13, 63]. However, this battery failed after only 104 cycles (Fig. S18a–b), most possibly because of the dendrite-induced short-circuit failure. Even before failure, the battery had already demonstrated very low CEs (85.9% in average), due to the parasitic reactions including I_3_^−^ shuttling and Zn corrosion/passivation. On the other hand, the battery with a Zeolite-Zn anode achieves a higher initial capacity of 203.0 mAh g^−1^ (Fig. 4c), which slightly decreased to 196.0 mAh g^−1^ after 500 cycles (96.6% capacity retention). The dramatically improved performance can be safely attributed to the effective suppression of parasitic reactions by the zeolite-based protecting layer, considering the high CEs (98.53% in average, Fig. 4d).

Since I_3_^−^ shuttling is the main reason accounting for self-discharge of Zn||I_2_ batteries, the I_3_^−^-blocking protecting layer should also be able to improve shelf life of the batteries. As exhibited in Fig. 4e, the bare-Zn battery loses 12.2% and 49.1% of its capacity after 10 and 50 h open-circuit resting, respectively, due to the fast consumption of the shuttling I_3_^−^ by the metallic Zn anode [23]. On the contrary, the Zeolite-Zn battery loss only 17.0% of its initial capacity after 50 h resting, indicating a nearly 3 times slower self-discharge rate (Fig. 4f). At a high rate of 2 A g^−1^, the Zeolite-Zn battery demonstrates extraordinary cycling stability (91.92% capacity retention after 5600 cycles) and CEs (99.76% in average, Fig. 4g), corresponding to an extremely slow capacity decay rate of 0.0016% per cycle, whereas the bare-Zn battery delivers not only low CEs (97.09% in average), but also short cycling lifetime (failed at 1015 cycles). The long battery lifetime enabled by the zeolite-based protecting layer has also been readily achieved in the Zn(AC)_2_ electrolyte, indicating the excellent reproducibility of this strategy (Fig. S19).

The achievement of stable and high areal capacity is another necessary precondition for practical application of battery systems. To highlight the application potential of this strategy, we further constructed a Zeolite-Zn||I_2_ full battery with an ultrahigh I_2_ mass loading of 13.3 mg cm^−2^ on the cathode. At a current density of 0.2 A g^−1^, this battery achieves an initial specific capacity of 134.2 mAh g^−1^ (areal capacity: 3.6 mAh, Fig. S20), corresponding to a Zn utilization coefficient of 7.2%. The capacity keeps almost unchanged within a testing period of 950 cycles. We further test the batteries connected in series or in parallel to mimic practical application conditions. At all conditions, the batteries deliver expected capacity and voltage outputs (Fig. S21).

## Conclusion

In summary, we develop a high performance, low-cost and intrinsically safe rechargeable Zn||I_2_ aqueous batteries, by means of comprehensively suppressing parasitic reactions on the Zn anodes with a zeolite-based cation-exchange protecting layer. On the one hand, the multifunctional zeolite-based layer allows smooth crossover migration of Zn^2+^, which Zn is deposited uniformly and rapidly. One the other hand, zeolite-based cation-exchange protecting layer can effectively block electrolyte and anions from passing through, and effective inhibit dendrite growth, Zn corrosion/passivation, and self-discharge. Thanks to the multifaceted merits of this protecting layer, the resulting Zeolite-Zn||I_2_ battery simultaneously achieves a high capacity (203–196 mAh g^−1^ at 0.2 A g^−1^), a high CE (99.76% in average at 2 A g^−1^), a long-term cycling stability (91.92% capacity retention after 5600 cycles at 2 A g^−1^). This work provides a new approach for the achievement of high-performance aqueous Zn||I_2_ batteries.

## Supplementary Information

Below is the link to the electronic supplementary material.

Supplementary file1 (PDF 2295 KB)
