# Supplementary material for: Boosting Zn||I2 Battery’s Performance by Coating a Zeolite-Based Cation-Exchange Protecting Layer
Source: Nanomicro Lett. 2022 Mar 25;14:82. doi: 10.1007/s40820-022-00825-5 (PMC8956761; doi:10.1007/s40820-022-00825-5)
Supplement: Supplementary file 1 — Supplementary file1 (PDF 2295 KB) [file 40820_2022_825_MOESM1_ESM.pdf]

Supporting Information for

Boosting Zn||I₂ Battery's Performance by Coating a Zeolite-Based Cation-Exchange Protecting Layer

Wenshuo Shang[1, #], Qiang Li[2, #], Fuyi Jiang[1, *], Bingkun Huang[1], Jisheng Song[1], Shan Yun[3], Xuan Liu[1], Hideo Kimura[1], Jianjun Liu[2, *], Litao Kang[1, *]

[1]College of Environment and Materials Engineering, Yantai University, Yantai 264005, P. R. China

[2]State Key Laboratory of High-Performance Ceramics and Superfine Microstructure, Shanghai Institute of Ceramics, Chinese Academy of Sciences, Shanghai 200050, P. R. China

[3]Key Laboratory for Palygorskite Science and Applied Technology of Jiangsu Province, Huaiyin Institute of Technology, Huai'an 223003, P. R. China

[#]W.S. Shang and Q. Li contributed equally to this paper and are co-first authors.

*Corresponding authors. E-mail: fyjiang@ytu.edu.cn (F.Y. Jiang); jliu@mail.sic.ac.cn (J. Liu); kanglitao@ytu.edu.cn (L.T. Kang)

Supplementary Figures and Table

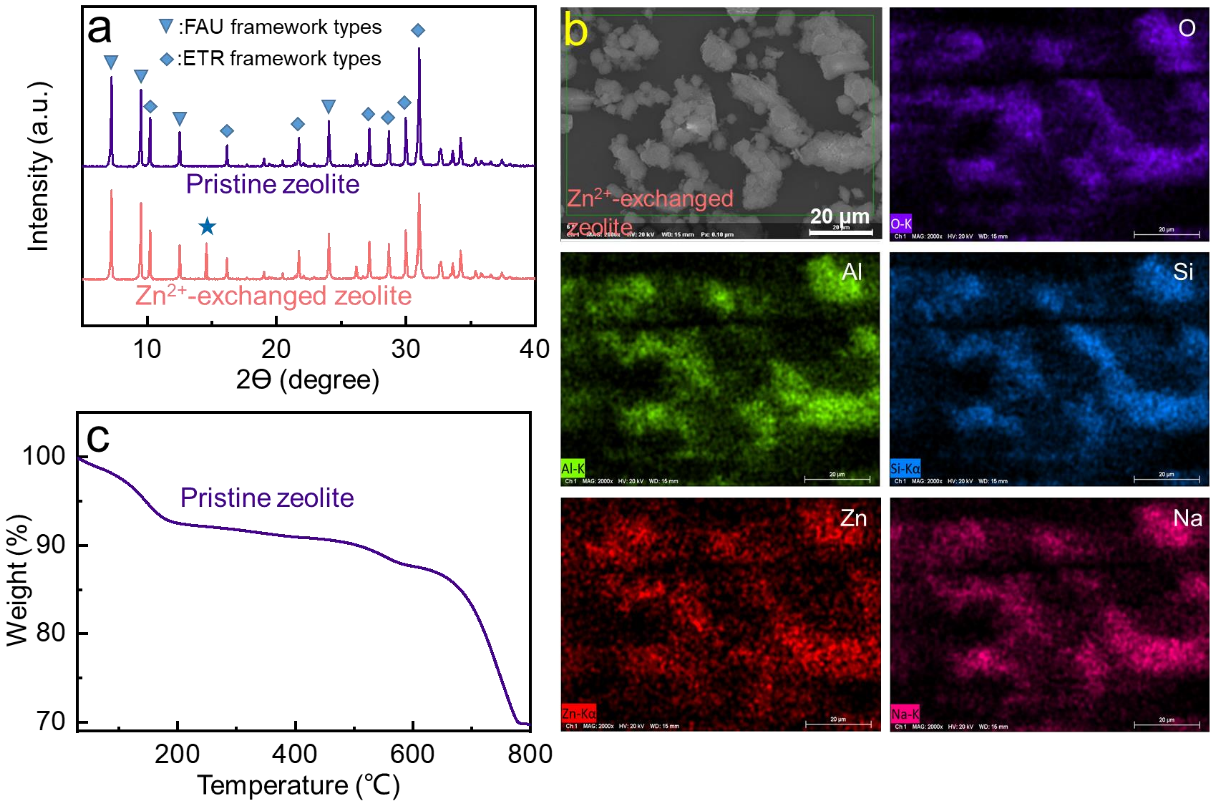

Fig. S1 a XRD patterns, **b** SEM and EDS elemental mapping, **c** thermogravimetric (TG) curve of the pristine or Zn^{2+}-exchanged zeolite.

As evidenced by XRD analyses (Fig. S1a), the employed zeolite powder consists of FAU framework types (JCPDS No. 38-0241) and ETR framework types (JCPDS No. 71-1557) compounds, according to the classification recommended by International Zeolite Association

(IZA) (C. Baerlocher and L.B. McCusker, Database of Zeolite Structures: http://www.iza-structure.org/databases/). The Si/Al molar ratio of this zeolite powder is determined to be 6.1 by using the inductively coupled plasma optical emission spectroscopy (ICP-OES) measurement. After 6 h soaking in 1M $ZnSO_4$ solution, a new XRD peak appears at 14.6°, due to the successful exchange of Na^+ with Zn^{2+} (Fig. S1b). TG test confirms the high thermal stability of the zeolite up to 500 °C (Fig. S1c). The weight loss from 50 to 200 °C can be attributed to the release of adsorbed and crystal water.

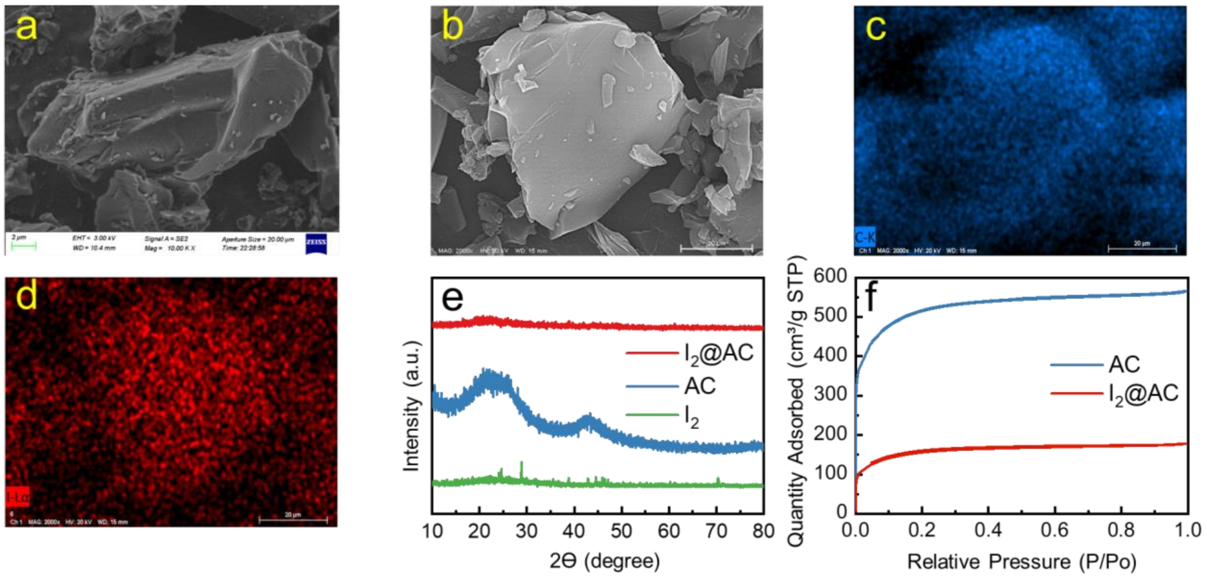

Fig. S2 SEM and EDS image of **a** activated carbon and **b-d** I_2@AC. **e** XRD patterns of I_2, the activated carbon matrix and the I_2@AC composite. **f** N_2 adsorption and pore size distributions isothermal of the activated carbon matrix and the I_2@AC composite

Figure S2a-d show that the iodine (I_2) had been uniformly infused into the activated carbon (AC) matrix in the I_2@AC composite cathode. After loading, the I_2 transformed from crystalline into amorphous (Fig. S2e), reflecting strong interactions between the matrix and the infused I_2. The bare AC matrix possesses a high BET quantity adsorbed of 566.2 cm^3 g^{-1}, which decrease down to 178.7 cm^3 g^{-1} after I_2-loading (Fig. S2f). These BET results also indicate the successful penetration of I_2 into the pores of the AC matrix.

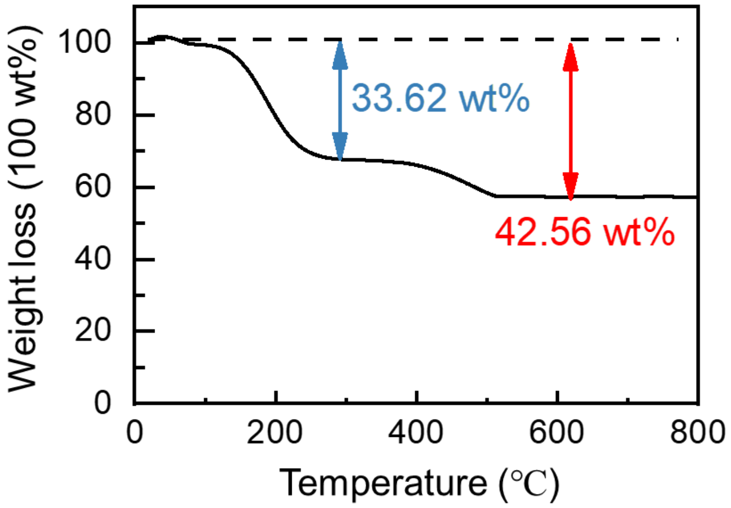

Fig. S3 TG curves of the dried I_2@AC cathode slurry in a dynamic nitrogen atmosphere within 30 ~ 800 °C

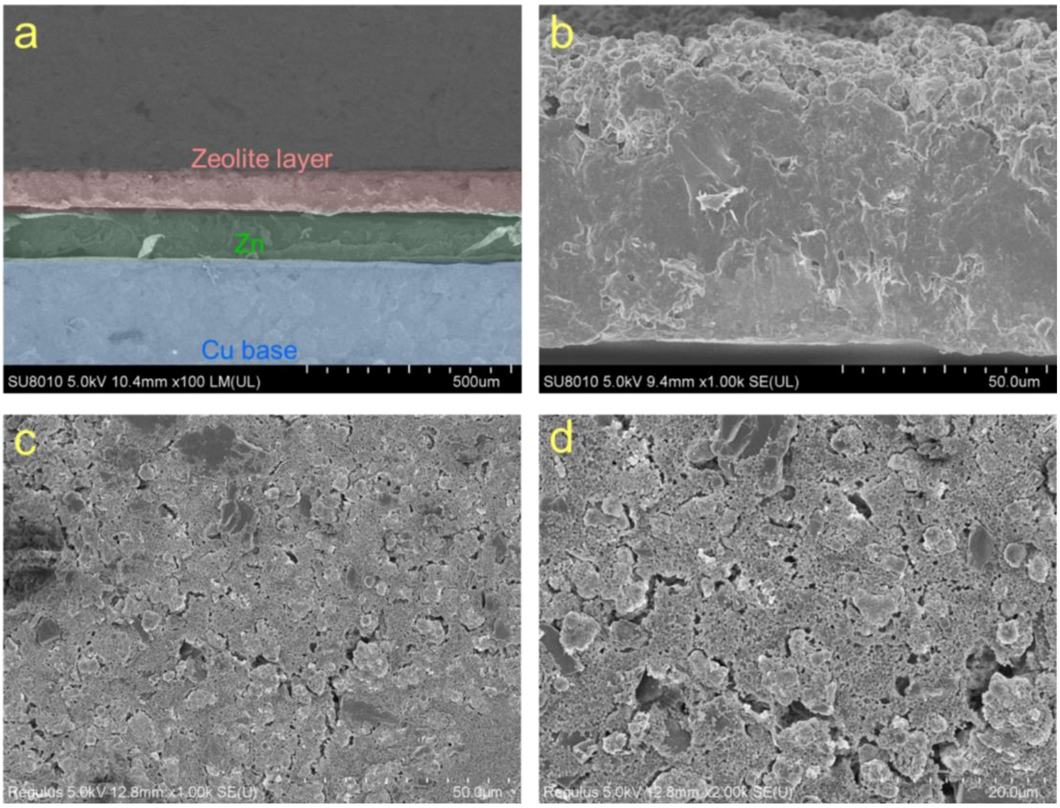

Fig. S4 a, b The cross-sectional and **c, d** top-view SEM image of Zn-based zeolite coating Zn foil

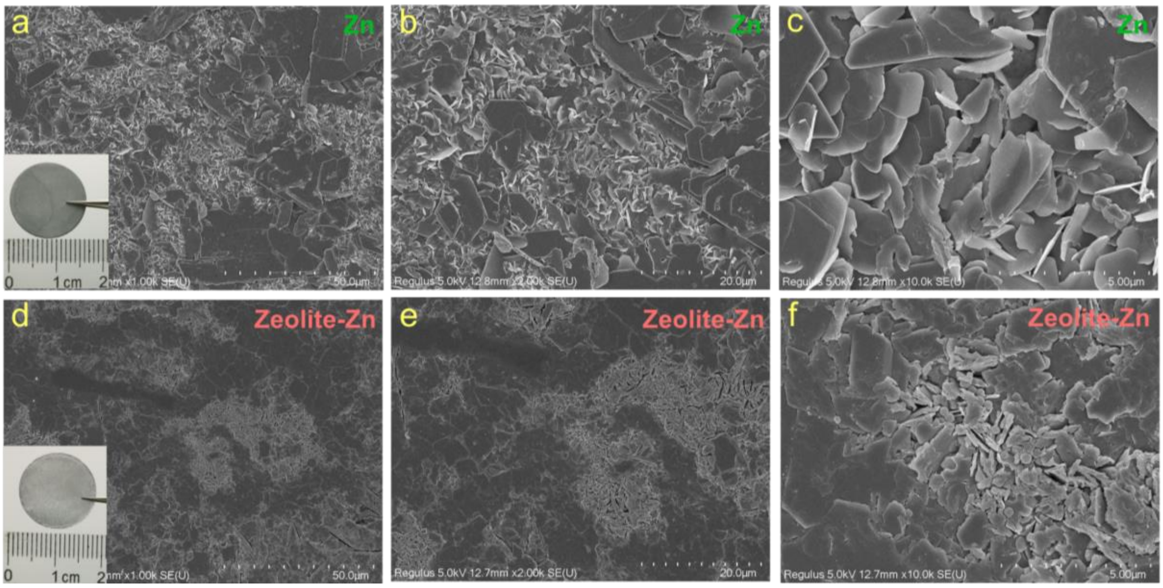

Fig. S5 SEM images of statically corroded **a-c** bare Zn or **d-f** Zeolite-Zn in 1 M $ZnSO_4$ electrolyte

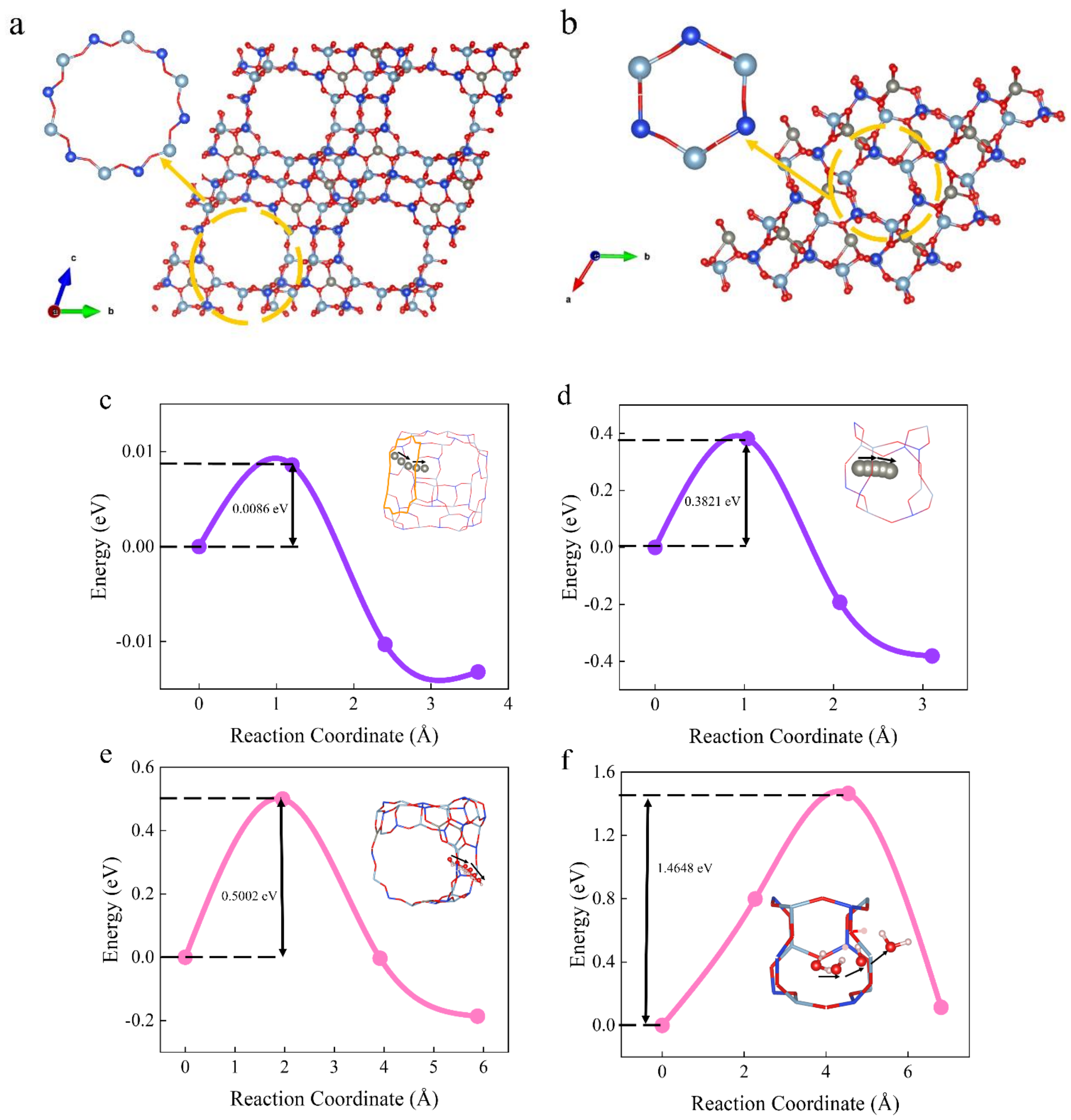

Fig. S6 Structure of **a** FAU and **b** ETR type zeolite constructed and optimized using the first principles[7]. DFT calculation of migration barrier of **c, d** Zn^{2+} and **e, f** hydrate Zn^{2+} within **c, e** FAU framework and **d, f** ETR framework zeolite lattice

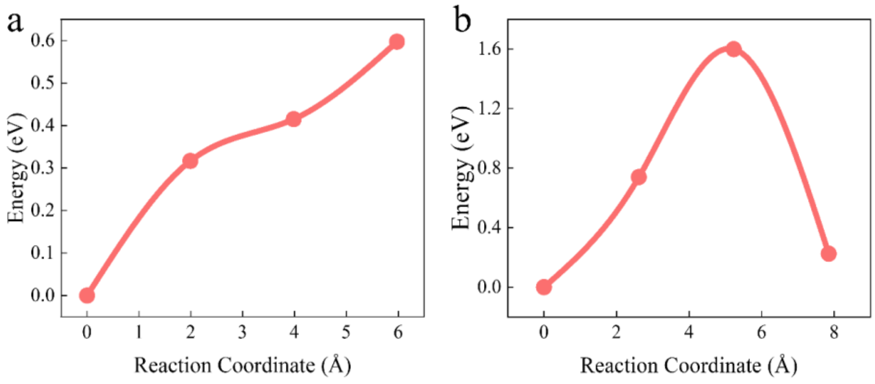

Fig. S7 DFT calculation of migration barrier of **a** I^{3-} and **b** hydrate I^{3-} within FAU framework

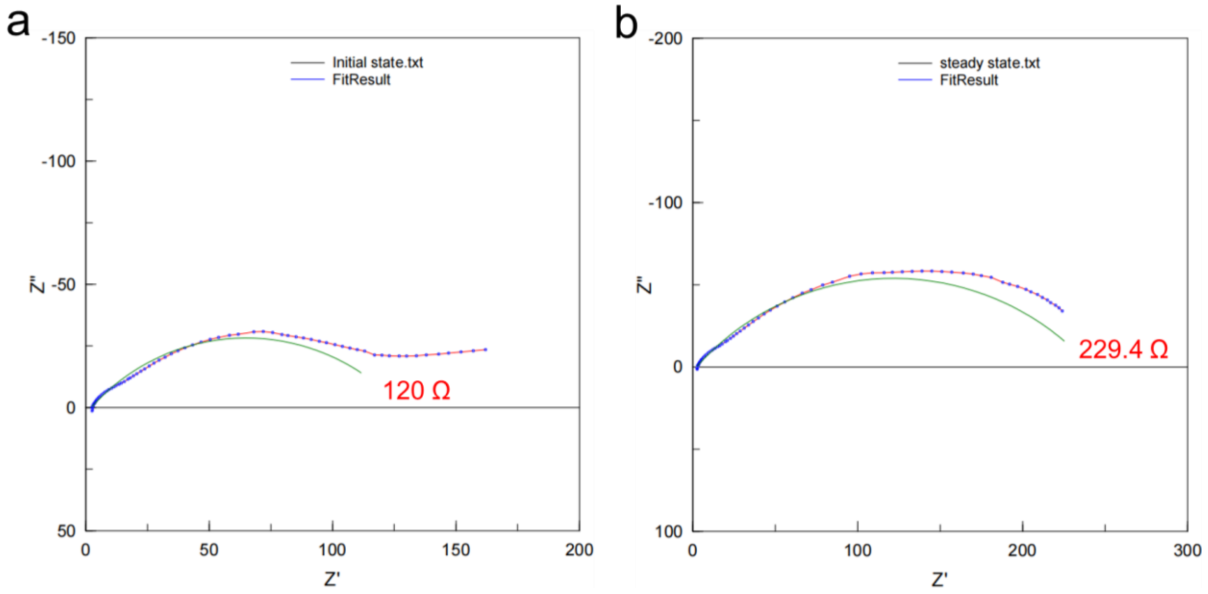

Fig. S8 Fitting results of the a initial and b steady state EIS plots showing in Fig. 2

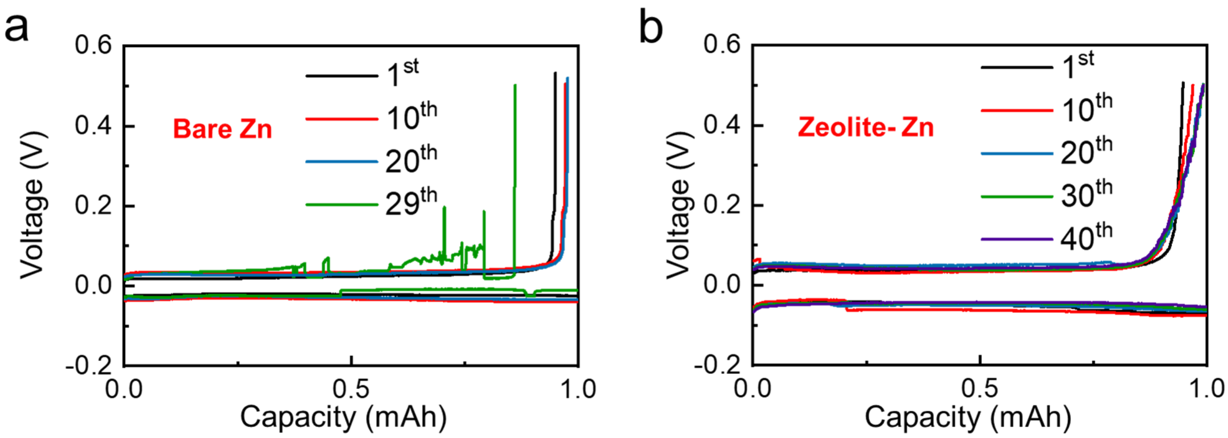

Fig. S9 Voltage profiles of the **a** bare Zn//Cu and **b** Zeolite-Zn//Zeolite-Cu asymmetric cells with 1 M $ZnSO_4$ electrolyte at a constant Zn striping/plating current density of 0.5 mA cm^{-2}. The corresponding Columbic efficiencies of these striping/plating processes are shown in Fig. 2d in the main text

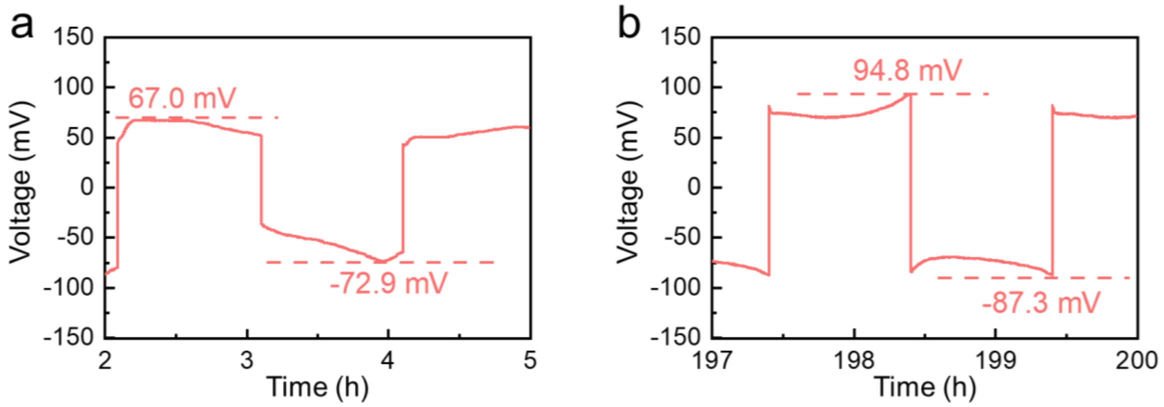

Fig. S10 Enlarged voltage profiles of Zeolite-Zn||Zeolite-Zn symmetric cells under the test condition shown in Fig. 2e

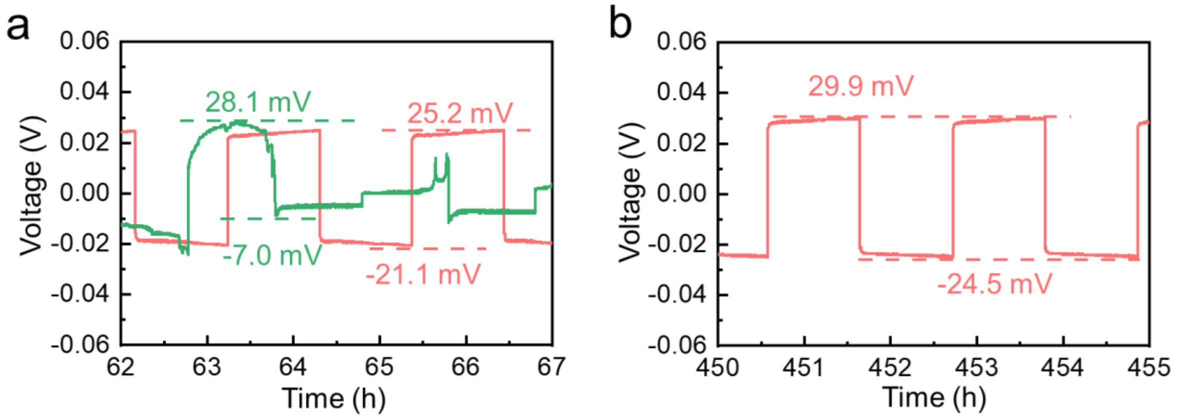

Fig. S11 The different cycle overpotential: **a** 50 - 51 cycles, **b** 250-251 cycles

The depth of discharge (DOD) of the symmetric cells was calculated by following equation:

$$DOD = \frac{3.6XM}{\rho N_A nel} \times 100\% \qquad \text{(S1)}$$

ρ represents zinc density ***ρ***= 7.14 g cm^{-3};

n represents the number of electrons lost for Zn0 converting to Zn^{2+} ***n***= 2;

M represents relative molecular mass of Zn ***M***= 65.4 g mol^{-1};

e represents electric quantity of a charge = 1.6×10^{-19} C;

l represents the thickness of Zn foil ***l***= 0.045 mm = 0.0045 cm;

N_A represents Avogadro constant ***N_A***= 6.02×10^{23};

X represents the capacity in each half cycle during charge/discharge. The deep discharge tasting capacity is 2.5 mAh cm^{-2};

With above parameters, the DOD of this condition is determined to be 9.51%.

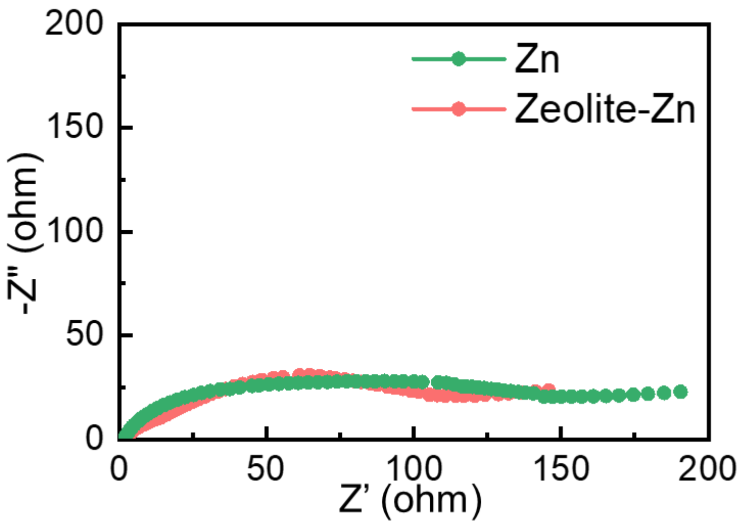

Fig. S12 Nyquist plots of the symmetric cells with either Zn or Zeolite-Zn electrodes

Table S1 Fitting results of the Nyquist plots using the equivalent circuit

Electrode	R_s (Ω cm^{-2})	CPE_1 (S s$^{1/2}$ cm^{-2})	CPE_2 (S s$^{1/2}$ cm^{-2})	R_{ct} (Ω cm^{-2})	W (S s$^{1/2}$ cm^{-2})
Bare-Zn	1.41	3.57×10^{-4}	0.64	157.26	0.52
Zeolite-Zn	2.32	2.45×10^{-3}	0.53	140.50	0.43

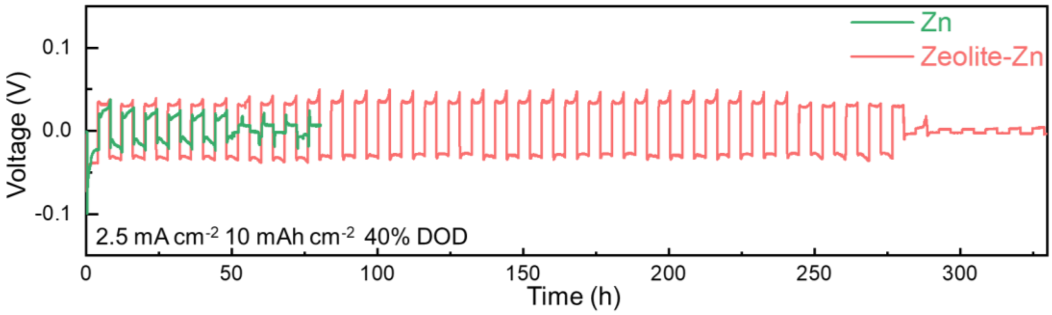

Fig. S13 GCD cycling tests of symmetric Zn||Zn cells at a large DOD of 40%

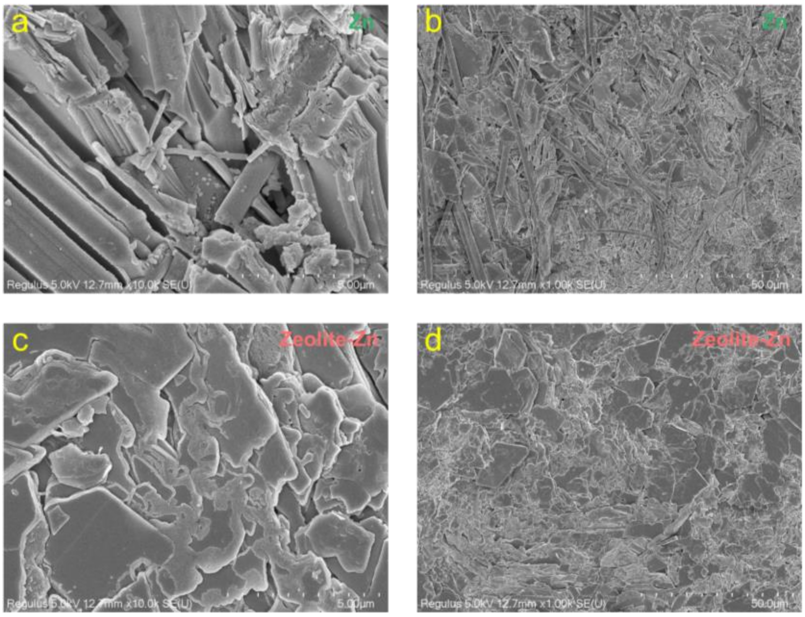

Fig. S14 Surficial SEM images of the **a, b** bare-Zn and **c, d** Zeolite-Zn electrodes after cycling test. To clearly show the morphology of underneath Zn, the zeolite layer on the Zeolite-Zn had been peeled off before SEM observation

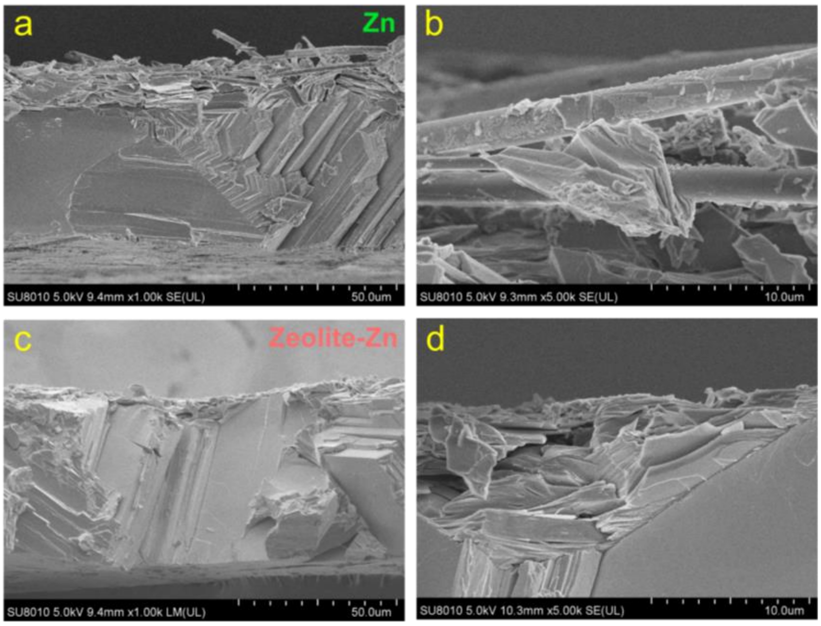

Fig. S15 Cross sectional SEM images of Zn foils in the bare- and Zeolite-Zn electrodes after cycling test. On the surface of the bare Zn electrolyte, there are obviously detaching debris that indicate the formation of "dead Zn"

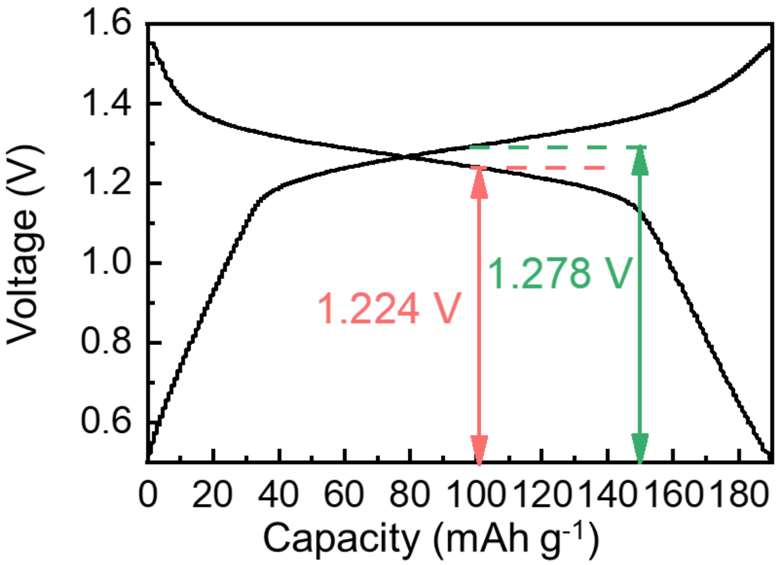

Fig. S16 Typical GCD curves of the Zeolite-Zn||I_2 battery at 0.1 A g^{-1}. The green and reds lines with arrows show the charge and discharge medium voltages (1.278 and 1.224 V, respectively)

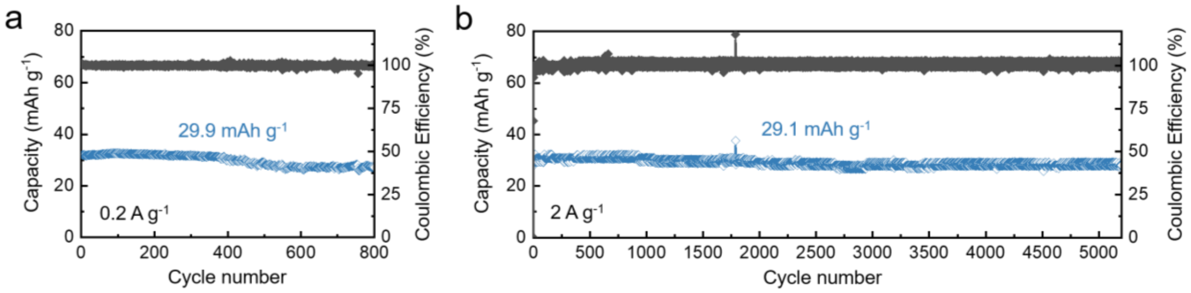

Fig. S17 Cycling performance of a pure activated carbon cathode with Zeolite-Zn anode at a current density of **a** 0.2 A g^{-1} and **b** 2 A g^{-1}

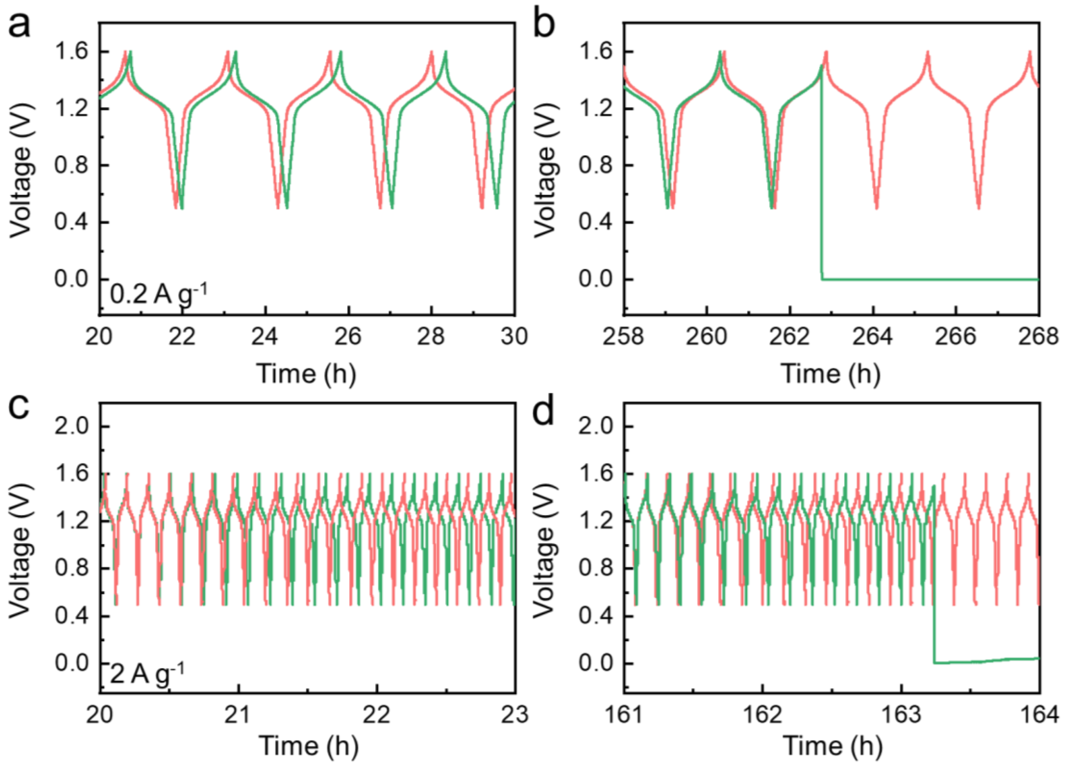

Fig. S18 The voltage - time curves of Zn-I_2 battery failure with **a, b** 0.2A g^{-1} and **c, d** 2 A g^{-1}

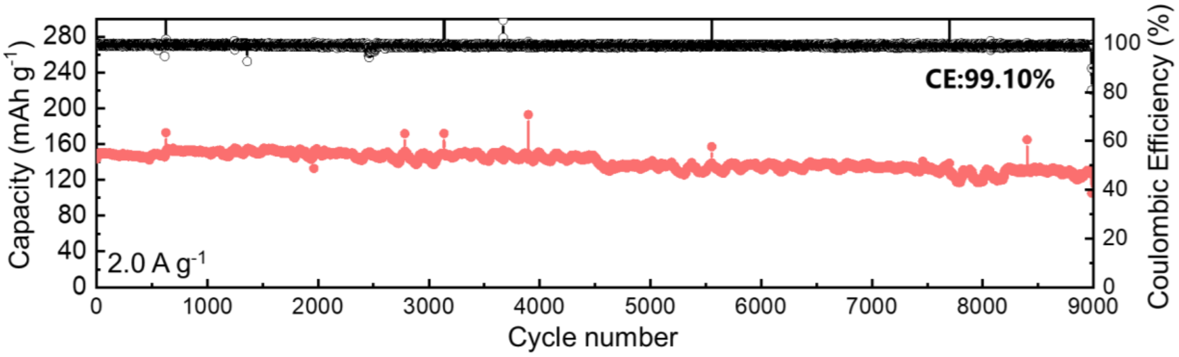

Fig. S19 Cycling performance and Coulombic efficiencies (CEs) of the Zeolite-Zn-I_2 battery with 1 M $Zn(Ac)_2$ at a current density of 2 A g^{-1}

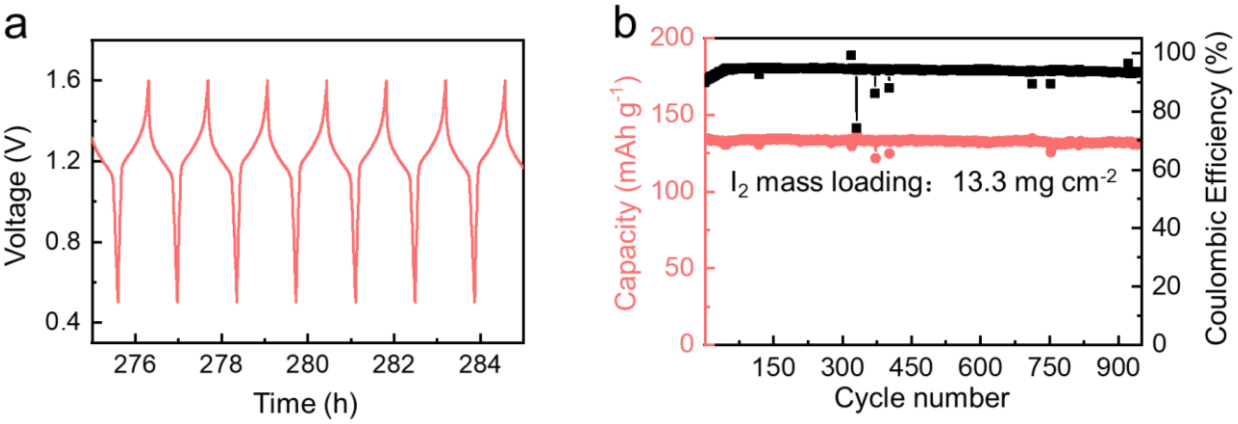

Fig. S20 a The voltage - time curves (200-207 cycles) and **b** cycling performance of Zeolite-Zn-I_2 batteries with 13.3 mg cm^{-2} I_2 mass loading at 0.2 A g^{-1}

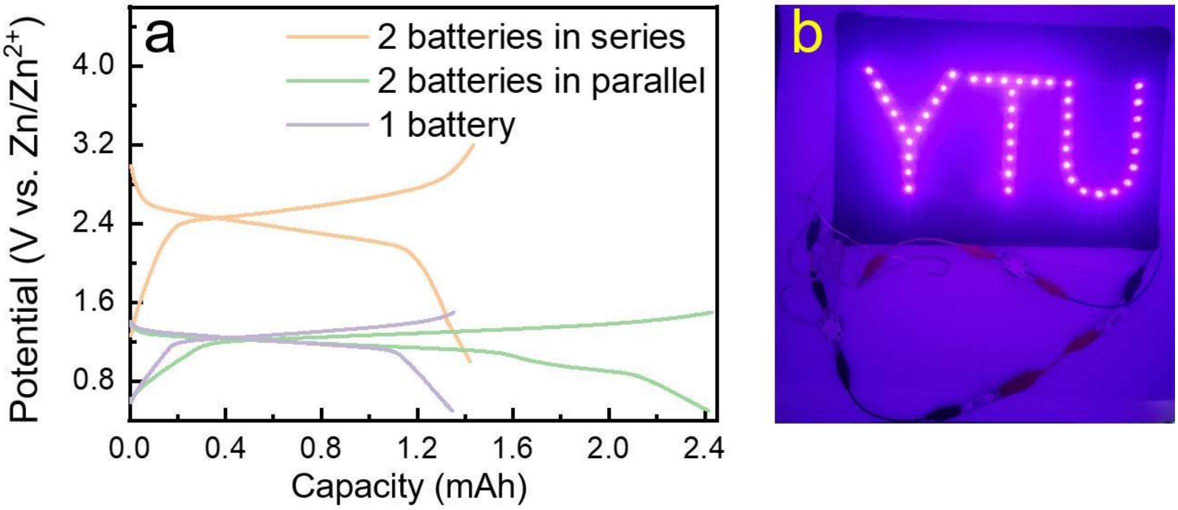

Fig. S21 a Charge/discharge curves of Zn||I_2 batteries connected in different way. **b** A LED light board powered by four Zn||I_2 batteries in series connection

The voltage and capacity output can be facilely tailored through battery connections. A single battery delivers a voltage of 1.22 V. Connecting two batteries in series or in parallel can double the voltage or capacity, respectively.

Supplementary References

[S1] C. Bai, F. Cai, L. Wang, S. Guo, X. Liu et al., A sustainable aqueous Zn-I_2 battery. Nano Res. **11**(7), 3548-3554 (2018). https://doi.org/10.1007/s12274-017-1920-9

[S2] W. Li, K. Wang, K. Jiang, A high energy efficiency and long life aqueous Zn-I_2 battery. J. Mater. Chem. A **8**(7), 3785-3794 (2020). https://doi.org/10.1039/C9TA13081K

[S3] K. Okhotnikov, T. Charpentier, S. Cadars, Supercell program: a combinatorial structure-generation approach for the local-level modeling of atomic substitutions and partial occupancies in crystals. J. Cheminform. **8**(1), 17 (2016). https://doi.org/10.1186/s13321-016-0129-3

[S4] P.E. Blöchl, Projector augmented-wave method. Phys. Rev. B **50**(24), 17953-17979 (1994). https://doi.org/10.1103/PhysRevB.50.17953

[S5] J.P. Perdew, K. Burke, M. Ernzerhof, Generalized gradient approximation made simple. Phys. Rev. Lett. **77**(18), 3865-3868 (1996). https://doi.org/10.1103/PhysRevLett.77.3865

[S6] G. Kresse, J. Furthmüller, Efficient iterative schemes for ab initio total-energy calculations using a plane-wave basis set. Phys. Rev. B **54**(16), 11169-11186 (1996). https://doi.org/10.1103/physrevb.54.11169

[S7] H. Cao, X. Cheng, H. Zhang, Beryllium carbide as diffusion barrier against Cu: first-principles study. Chin. Phys. B **29**(1), 016601 (2020). https://doi.org/10.1088/1674-1056/ab593e
